# Case Report: Progressive acquired ileal mesenteric venous malformation after right hemicolectomy: serial imaging evolution leading to delayed gastrointestinal bleeding

**DOI:** 10.3389/fsurg.2026.1883796

**Published:** 2026-07-13

**Authors:** Deqi Wu, Songyang Wu, Yao Luo, Jinyuan Yu, Zhenguang Chen

**Affiliations:** 1 Department of Thyroid and Breast Diagnosis and Treatment Center, Shulan (Hangzhou) Hospital, Shulan International Medical College, Zhejiang Shuren University, Hangzhou, China; 2 Department of Gastrointestinal and Anorectal Diagnosis and Treatment Center, Shulan (Hangzhou) Hospital, Shulan International Medical College, Zhejiang Shuren University, Hangzhou, China

**Keywords:** acquired mesenteric venous malformation, delayed gastrointestinal bleeding, ileal bleeding, mesenteric venous hypertension, postoperative vascular remodeling, right hemicolectomy

## Abstract

**Background:**

Mesenteric venous malformations are rare vascular anomalies. Acquired lesions involving the gastrointestinal mesentery are particularly uncommon and may present years after abdominal surgery with obscure gastrointestinal bleeding, posing a diagnostic challenge.

**Case presentation:**

We report a rare case of progressive acquired ileal mesenteric venous malformation developing after right hemicolectomy. A 50-year-old woman underwent curative right hemicolectomy for colon cancer in 2016. Four years later, she presented with recurrent painless hematochezia and anemia, while colonoscopy failed to identify the bleeding source. Contrast-enhanced CT angiography demonstrated clusters of dilated and tortuous veins within the ileal mesentery, forming a caput medusae–like appearance and draining into the superior mesenteric vein, with additional communication to the lumbar vein. Exploratory surgery confirmed a localized venous plexus along the mesenteric border of the terminal ileum. Targeted ligation and suturing of the abnormal vessels resulted in complete resolution of bleeding.

**Conclusion:**

Acquired ileal mesenteric venous malformation should be considered in patients with unexplained delayed gastrointestinal bleeding after right hemicolectomy. This case uniquely documents progressive lesion evolution on serial imaging, supporting the hypothesis that postoperative venous outflow alteration and chronic venous remodeling may contribute to lesion development. Recognition of this entity may facilitate timely diagnosis and surgical management.

## 1 Introduction

Venous malformations (VMs) are one of the most common congenital vascular malformations, with low-flow vascular anomalies characterized by dysplastic and dilated venous channels (1). The prevalence of VMs is about 1%–1.5% and the incidence rate is 1–5 per 10,000 (2–4). About 40% of lesions are found in the head and neck, 40% in the extremities, and 20% in the torso (5, 6). Intra-abdominal VMs are rare, and involvement of the gastrointestinal tract is even less common. It can occur anywhere in the digestive tract (esophagus, stomach, small or large intestine, anus) (7). Among these, mesenteric VMs represent an exceptionally rare subset.

Mesenteric VMs may be congenital or acquired (3, 4). Congenital lesions are usually present early in life, whereas acquired lesions are typically associated with abdominal trauma, inflammation, or prior intestinal surgery (8). Importantly, acquired mesenteric VMs may remain asymptomatic for years and present long after the inciting event. When clinical manifestations occur, gastrointestinal bleeding is the most common, accounting for 2.6%–6.2% of gastrointestinal bleeding cases, usually resulting from involvement of the bowel wall and rupture of ectatic submucosal veins (9).

We report a rare case of progressive acquired ileal mesenteric VMs with lumbar vein collateral formation, developing several years after right hemicolectomy and ultimately leading to lower gastrointestinal bleeding. This case highlights the underlying role of postoperative venous hemodynamic alteration in the pathogenesis of acquired mesenteric VMs.

## 2 Key clinical messages

- Acquired mesenteric VMs are extremely rare and may occur years after intestinal surgery due to postoperative alterations in venous outflow.
- Serial imaging in this case captured the gradual evolution of abnormal mesenteric venous structures that were absent before surgery, providing rare evidence of postoperative vascular remodeling.
- Acquired ileal mesenteric VMs should be considered in patients presenting with unexplained delayed gastrointestinal bleeding after right hemicolectomy.
- Careful review of longitudinal imaging can facilitate early diagnosis and guide targeted surgical management.

## 3 Case presentation

A 50-year-old female patient underwent curative right hemicolectomy for colon cancer in November 2016. The postoperative course was unremarkable. Follow-up abdominal CT in September 2017 demonstrated no vascular abnormalities.

Approximately 4 years postoperatively, she began experiencing intermittent painless melena. No weight loss was observed. Laboratory evaluation revealed anemia, and colonoscopy failed to identify a definite bleeding source. The rest of the systemic examination was normal. After that, the illness recurred repeatedly and lasted for more than 5 months.

**Figure 1:**
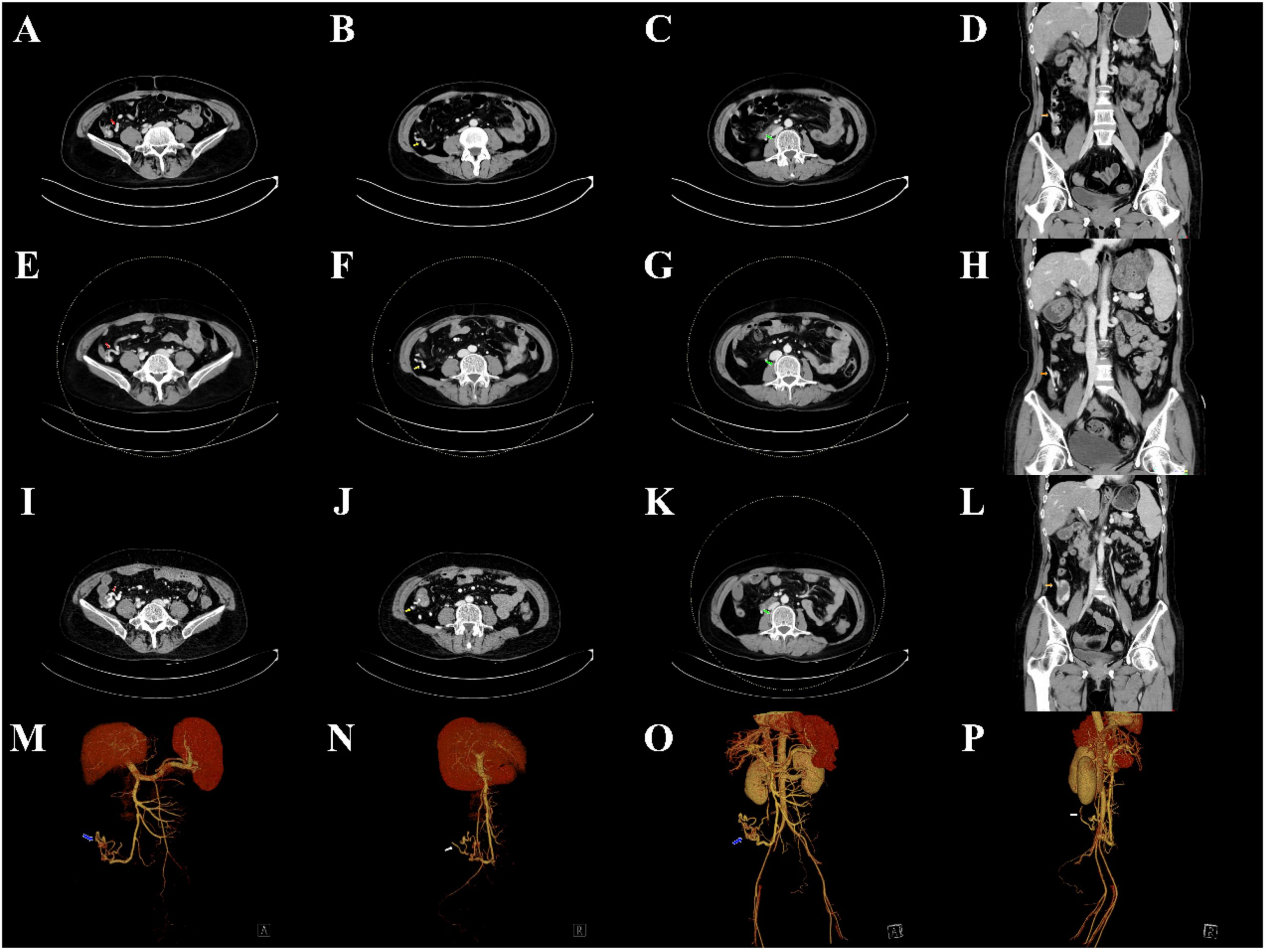
Imaging findings obtained on March 25, 2021 **(A–D)**, May 2, 2021 **(E–H,M,N)**, and August 27, 2021 **(I–L,O,P)**. Clusters of abnormally dilated and tortuous vessels were identified within the ileal mesentery (yellow arrows), exhibiting a caput medusae–like appearance (blue arrows). These vessels drained into tributaries of the superior mesenteric vein (red arrows). Abnormal venous communication with the inferior vena cava and/or the right lumbar vein was observed at the L4–L5 level (green and white arrows). Serial imaging demonstrated progressive enlargement and increasing vascular complexity of the lesion over the 5-month observation period (orange arrows), supporting the diagnosis of a progressively evolving acquired mesenteric VMs. An additional movie file provides further visualization of these findings (see Supplementary Files S5–S7).

**Figure 2:**
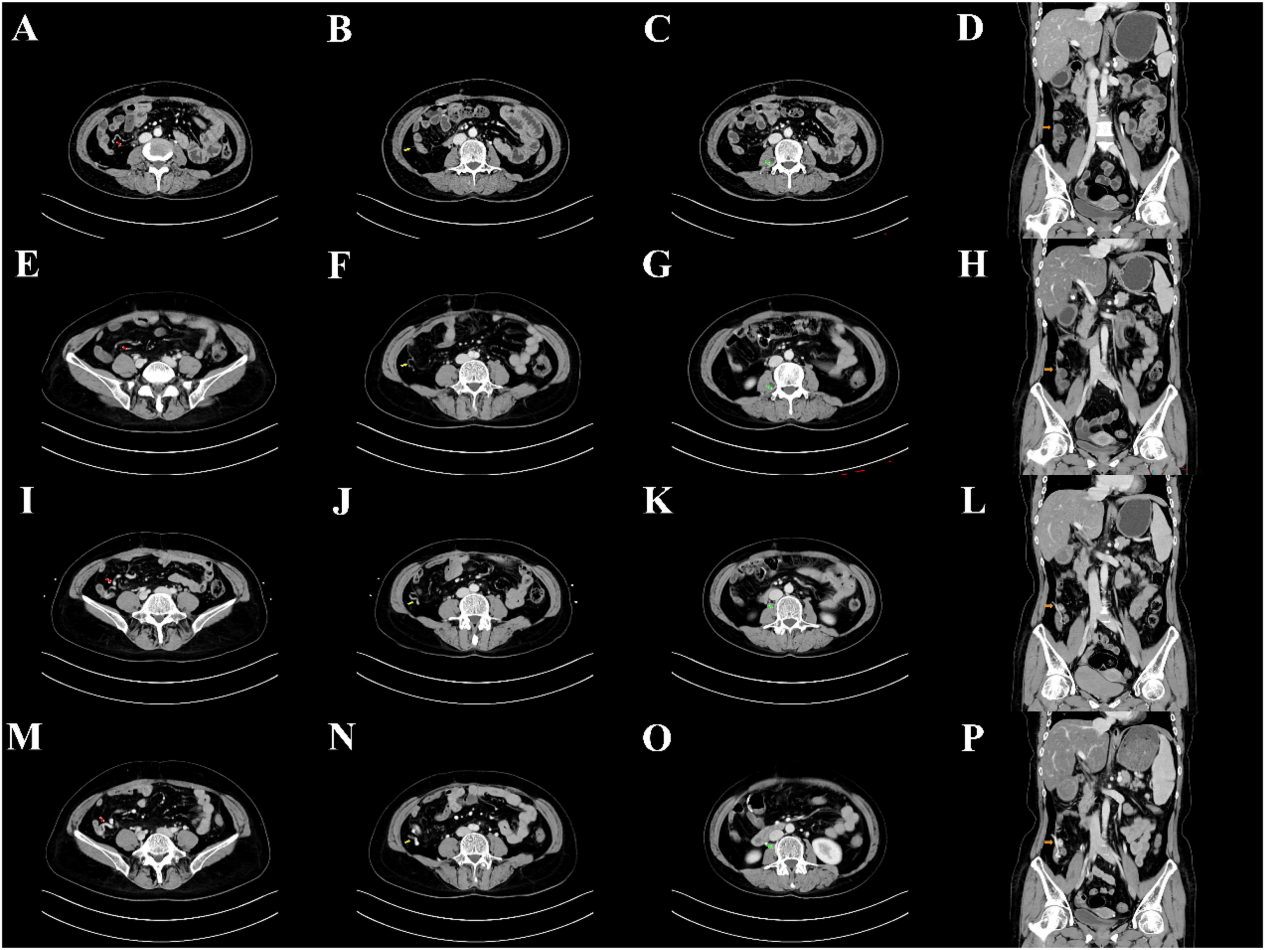
Retrospective review of prior imaging demonstrated a progressive evolution of the lesion: imaging findings on September 13, 2017: No vascular abnormality was observed **(A–D)**; on June 9, 2018 **(E–H)** and March 22, 2019 **(I–L)**: abnormally dilated and tortuous vessels began to appear and gradually deteriorated. On July 7, 2020, the morphological complexity of the blood vessels increased **(M–P)**, and lumbar communicating branches were formed (green arrows). The tributaries of the superior mesenteric vein (red arrows); dilated and tortuous vessels (yellow arrows); venous communication with the inferior vena cava and/or the right lumbar vein (yellow arrows); lesions (orange arrows);An additional movie file shows this in more detail (see Supplementary Files S1–S4).

On August 27, 2021, contrast-enhanced abdominal CT angiography revealed three clusters of abnormally dilated and tortuous vessels along the ileal mesentery, forming a caput medusae–like appearance. These vessels converged into the superior mesenteric vein, and abnormal venous communication with the inferior vena cava or right lumbar vein was observed at the L4–L5 level (Figures 1A–P). Portal venous phase imaging demonstrated contrast extravasation into the intestinal lumen, which increased on delayed phases. Retrospective review of prior imaging demonstrated a progressive evolution of the lesion: no abnormality in 2017, subtle venous dilation in 2018, and gradual enlargement and increased complexity in 2019 and 2020 (Figures 2A–P). Notably, the lesion was absent on preoperative imaging. The maximal diameter of the abnormal venous plexus increased progressively from approximately 3.3 mm in 2018 to 4.5 mm in 2019, 5.1 mm in 2020, and 6.0 mm in 2021. No early arterial enhancement was observed. Progressive opacification became evident during the portal venous and venous phase, and persisted on delayed-phase imaging, consistent with a low-flow venous lesion. Collateral venous drainage through a right lumbar venous channel at the L4–L5 level became increasingly conspicuous over time. The maximal diameter from approximately 3.1 mm in 2018 to 3.5 mm in 2019, 3.8 mm in 2020, and 4.3 mm in 2021.

Therefore, the patient underwent exploratory laparotomy. During surgery, an abnormal venous plexus was identified along the mesenteric border of the terminal ileum, approximately 50 cm proximal to the ileocolic anastomosis. Additional abnormal veins were seen entering the mesentery from the retroperitoneum. Suturing and ligating the malformed blood vessels was performed, and the patient recovered uneventfully with complete resolution of gastrointestinal bleeding.

**Figure 3:**
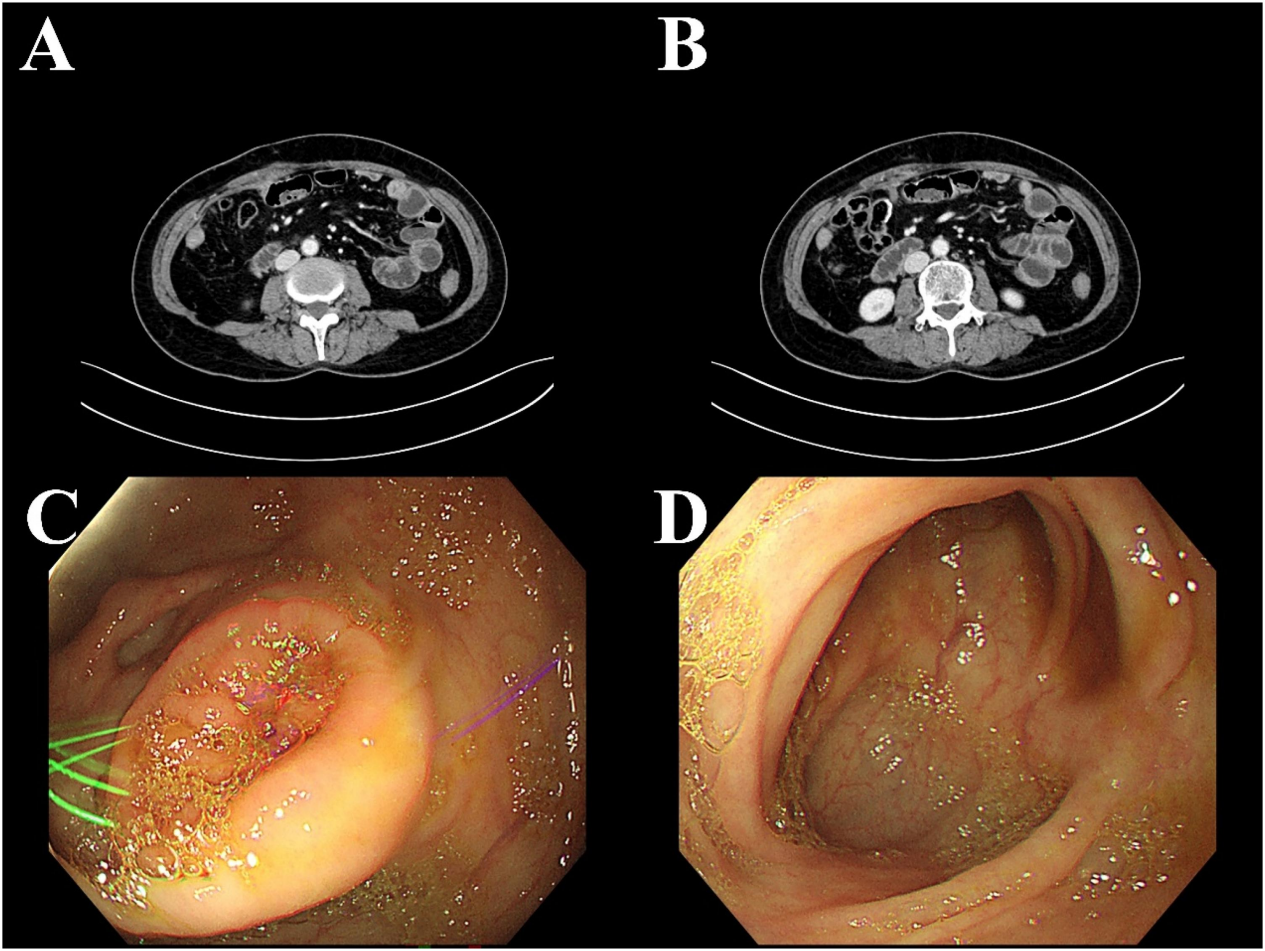
Post-treatment follow-up CT obtained at 48 months. The abnormal venous plexus seen on prior imaging was no longer visible, and lumbar collateral communications were substantially regressed **(A,B)** (see Supplementary File S8) Colonoscopy showed normal results without obvious abnormalities **(C,D)**.

At 58 months of postoperative follow-up, the patient remained asymptomatic without recurrent hematochezia. Hemoglobin levels remained stable at 110–128 g/L. Follow-up CT performed at 48 months demonstrated disappearance of the previously identified abnormal venous plexus and significant regression of lumbar collateral communication. (Figures 3A,B). Also, the colonoscopy findings were unremarkable (Figures 3C,D).

The evolution of the disease over time is summarized in Table 1.

**Table 1:** Clinical timeline.

Timeline	Clinical event
2016-11-28	Curative right hemicolectomy
2017-09-13	Regular post-operation follow-up
2018-06-09	Subtle venous dilation
2019-03-22	Tortuous vessels appear
2020-07-07	Abnormal lumbar communicating branches were formed
2021-03-25	Painless hematochezia and anemia
2021-05-02	Ileal mesenteric VMs
2021-08-30	Exploratory laparotomy
2025-08-09	48 Months follow-up CT angiography abnormal venous plexus dissappear

## 4 Discussion

### 4.1 Underlying pathogenetic mechanism of VMs formation in this case

The present case demonstrated a stepwise evolution of abnormal mesenteric venous structures over a 5-year period, characterized by progressive venous dilatation, increasing vascular complexity, and eventual development of lumbar collateral drainage pathways. The absence of preoperative vascular abnormalities together with the documented temporal progression on serial imaging favors an acquired venous lesion rather than a congenital vascular malformation. These observations suggest a dynamic remodeling process that developed after surgery rather than a pre-existing vascular anomaly.

Nevertheless, although the imaging findings are compatible with chronic adaptive remodeling secondary to altered venous hemodynamics, direct venous pressure measurements were unavailable, and therefore a causal relationship between right hemicolectomy and lesion formation cannot be definitively established. Importantly, right hemicolectomy is a common procedure, whereas similar lesions are exceedingly rare. This observation suggests that postoperative venous outflow alteration alone may not be sufficient for lesion development. Additional patient-specific factors, including individual variations in mesenteric venous anatomy, collateral drainage capacity, or other unidentified local hemodynamic influences, may also contribute. In the present patient, no evidence of portal hypertension, mesenteric venous thrombosis, systemic venous disease, or known thrombophilia was identified.

Chronic venous outflow impairment has been reported to induce venous dilatation, tortuosity, and collateral formation, as observed in small bowel varices and other conditions associated with mesenteric venous obstruction (8, 10, 11). In the present case, contrast-enhanced CT angiography demonstrated clusters of dilated and tortuous mesenteric veins converging into the superior mesenteric vein, together with abnormal venous communication at the L4–L5 level involving the inferior vena cava or right lumbar vein. The development of such lumbar venous collateralization may represent an adaptive decompressive response potentially associated with chronically altered venous outflow and localized venous hypertension. If sustained mesenteric venous hypertension develops, redistribution of venous flow through retroperitoneal channels, including lumbar veins, may occur because of their anatomical communication with both the mesenteric venous system and the inferior vena cava.

Beyond serving as a marker of chronic venous remodeling, progressive venous hypertension may also have clinically relevant consequences. Progressive involvement of submucosal venous channels may ultimately predispose patients to delayed gastrointestinal bleeding. Similar dilated, thin-walled venous or vascular structures within the mucosa and submucosa have been documented as sources of occult or overt gastrointestinal bleeding in vascular malformations (12, 13). Although histopathological confirmation of venous hypertension was unavailable in the present case, the temporal radiological progression and collateral venous development support the possibility that chronic postoperative alterations in venous drainage contributed to the evolution of this lesion.

### 4.2 Diagnostic considerations, imaging characteristics and differential diagnosis of mesenteric VM

Diagnosis of mesenteric VM is challenging due to their rarity and nonspecific clinical presentation. Endoscopic evaluation often fails to identify the bleeding source, particularly when the lesion is located in the small intestine. Despite the performance of conventional colonoscopy and capsule endoscopy in this case, the source of the patient's bleeding remained undetectable. Although double-balloon enteroscopy can be valuable for evaluating obscure small-bowel bleeding, this modality was not available at our institution at the time of the patient's evaluation, and therefore deep small-bowel endoscopic assessment could not be performed. This represents a limitation of the present report. In this case, contrast-enhanced CTA clearly demonstrates venous dilation, drainage routes and collateral pathways, providing key information for surgical planning and intraoperative hemostasis. Serial imaging is particularly valuable in distinguishing acquired lesions from congenital anomalies by demonstrating progressive postoperative development. Based on the existing classification of vascular anomalies, the lack of early venous filling in the arterial phase and marked delayed venous pooling indicate a low-flow VM instead of a high-flow arteriovenous malformation (14, 15). Our case, imaging showed venous dilatation and abnormal drainage without arterial feeders, a nidus, or early arteriovenous shunting, consistent with an acquired VM. Characteristic findings included caput medusae–like venous clusters, abnormal lumbar vein communications, and delayed-phase contrast extravasation into the bowel lumen, further supporting a venous-driven pathology.

Several alternative diagnoses were considered. Acquired mesenteric varices were an important differential diagnosis because the lesion developed after bowel surgery and was associated with chronic venous remodeling. However, the patient had no evidence of portal hypertension, portal venous thrombosis, mesenteric venous occlusion, or diffuse portosystemic collateralization. Moreover, serial imaging demonstrated progressive formation of a localized plexiform vascular network rather than simple dilation of a pre-existing venous channel. Postoperative angiodysplasia was considered less likely because angiodysplastic lesions are typically confined to the mucosal and submucosal vascular plexus and are rarely associated with extensive mesenteric venous enlargement or retroperitoneal collateral pathways. Likewise, neither an arteriovenous fistula nor a high-flow arteriovenous malformation was supported by imaging findings, as no enlarged feeding artery, nidus formation, early arterial-phase venous opacification, or arteriovenous shunting was identified.

Nevertheless, because histopathological confirmation was unavailable, definitive classification according to ISSVA criteria remains uncertain. Therefore, the lesion is most appropriately regarded as a presumed acquired mesenteric venous malformation-like lesion based on the available clinical, radiological, longitudinal imaging, and intraoperative findings.

### 4.3 Treatment considerations

Mesenteric VMs represent an uncommon subset of low-flow vascular anomalies, and optimal management remains challenging because of their rarity and heterogeneity. Treatment strategies should be tailored to clinical presentation, anatomical distribution, and the extent of bowel involvement, with options ranging from conservative observation to interventional radiology and surgery, often requiring multidisciplinary decision-making (16). Although endovascular or embolization techniques have been reported as effective in selected cases, particularly for complex or surgically inaccessible lesions, their role may be limited when the malformation involves the bowel wall or is associated with recurrent gastrointestinal bleeding (17). Surgical treatment therefore continues to play a central role in the management of symptomatic intestinal VMs. In patients with persistent or recurrent bleeding, surgery offers definitive control, especially when less invasive measures are ineffective or unsuitable (18). Importantly, the extent of surgical intervention should be carefully balanced against the risk of unnecessary bowel resection. When the lesion is localized and there is no evidence of bowel ischemia or extensive transmural involvement, limited procedures such as targeted ligation or suturing of abnormal venous structures may be adequate and align with established surgical principles for venous sources of gastrointestinal bleeding (18).

In the present case, the VM was confined, and intraoperative evaluation demonstrated preserved bowel perfusion without ischemic changes. Based on these findings, a limited surgical approach consisting of suturing and ligation of the malformed vessel was undertaken rather than segmental bowel resection. This strategy resulted in complete symptom resolution, highlighting that carefully selected patients with localized mesenteric VMs may benefit from targeted surgical management while avoiding unnecessary loss of bowel length.

### 4.4 Limitations and clinical implications

This report has several limitations. Histopathological confirmation was not available because the lesion was primarily characterized based on imaging and intraoperative findings. Nevertheless, the combination of serial imaging, characteristic venous features, and surgical observations provides strong evidence supporting the diagnosis.

Retrospective review demonstrated that the earliest vascular changes were subtle and not initially recognized as clinically significant. Awareness of progressive mesenteric venous remodeling on surveillance imaging may facilitate earlier diagnosis and intervention before the onset of clinically significant gastrointestinal bleeding. This case underscores the importance of considering acquired mesenteric VM in patients presenting with delayed gastrointestinal bleeding years after colorectal surgery and highlights the diagnostic value of longitudinal imaging review.

As this is a single case report, no definitive mechanistic inference can be drawn. The proposed association between postoperative venous outflow alteration and subsequent vascular remodeling should be interpreted as a hypothesis requiring validation in future studies.

## 5 Conclusion

Acquired ileal mesenteric VMs are a rare cause of delayed gastrointestinal bleeding after right hemicolectomy. Chronic postoperative venous hypertension, adaptive collateral formation, and ectatic remodeling likely underlie their development. Awareness of this entity and its hemodynamic pathophysiology is essential for early diagnosis and curative management.

## 6 Patient perspective

The following statement summarizes the patient's views as expressed during postoperative follow-up interviews and was reviewed and approved by the patient.

The patient initially experienced intermittent gastrointestinal symptoms that progressively worsened over time, eventually developing recurrent bleeding that significantly affected daily life and caused considerable anxiety. Multiple evaluations were required before the underlying mesenteric vascular lesion was fully recognized. After surgical ligation and suturing of the abnormal vessels, the symptoms completely resolved, and the patient was relieved to avoid bowel resection. The patient expressed satisfaction with both the recovery process and the long-term outcome after treatment.

## Data Availability

The original contributions presented in the study are included in the article/Supplementary Material, further inquiries can be directed to the corresponding author.
